# The *In vitro* Antibiotic Tolerant Persister Population in *Burkholderia pseudomallei* is Altered by Environmental Factors

**DOI:** 10.3389/fmicb.2015.01338

**Published:** 2015-12-15

**Authors:** William C. Nierman, Yan Yu, Liliana Losada

**Affiliations:** Infectious Diseases Program, J. Craig Venter Institute, La JollaCA, USA

**Keywords:** melioidosis, disease recurrence, antibiotic tolerance, persister fraction, microbiology, environmental signals

## Abstract

Bacterial persistence due to antibiotic tolerance is a critical aspect of antibiotic treatment failure, disease latency, and chronic or reemergent infections. The levels of persisters is especially notable for the opportunistic Gram-negative pathogens from the *Burkholderia* and *Pseudomonas* genera. We examined the rate of drug tolerant persisters in *Burkholderia pseudomallei, Burkholderia thailandensis, Burkholderia cepacia* complex organisms, and *Pseudomonas aeruginosa* at mid-log growth in LB broth culture. We found that a fraction of the antibiotic-sensitive cells from every species were tolerant to a 24 h high-dose antibiotic challenge. All tested *Burkholderia* strains demonstrated a drug tolerant persister population at a rate that was at least 100–500 times higher than *P. aeruginosa*. When challenged with at least a 10X minimum inhibitory concentration (MIC) 24 h exposure to three different antibiotics with different modes of action we found that in *B. pseudomallei* Bp82 each of the tree antibiotics revealed different persister fractions at each of two different growth states. This observation suggests that our assay is detecting heterogeneous persister subpopulations. Persistence in *B. pseudomallei* Bp82 was highly dependent on growth stage, with a surprisingly high persister fraction of >64% of the late stationary phase cells being antibiotic tolerant to 100XMIC cefotaxime. Adaptation of *B. pseudomallei* to distilled water storage resulted in a population of drug tolerant cells up to 100% of the non-drug-challenged viable cell count in the same cefotaxime assay. Cultivation of *B. pseudomallei* with a sub-inhibitory concentration of several antibiotics resulted in altered persister fractions within the population relative to cultures lacking the antibiotic. Our study provides insight into the sensitivity of the persister fraction within the population of *B. pseudomallei* due to environmental variables and suggests diversity within the persister population revealed by different challenge antibiotics.

## Introduction

The emergence of resistant organisms was reported soon after the introduction of antibiotics into clinical practice (Abraham and Chain, 1940). Similarly, the inability of antibiotic treatment to sterilize wounds thus resulting in recurrent infections after completion of the treatment was reported slightly later (Bigger, 1944). The ability of a small subpopulation of bacteria that survives a lethal antibiotic challenge is termed persistence or drug tolerance. This phenomenon is distinct from resistance in that once these drug tolerant cells resume normal growth after the drug is removed; they exhibit the same drug sensitivity as the population originally treated. Unlike resistance, persistence is not heritable. For reviews of persistence see (Lewis, 2010; Cohen et al., 2013). Increasing evidence suggests that drug tolerance or persistence is in fact an actively maintained state, triggered and enabled by a network of intracellular stress responses that result in drug tolerance in a fraction of the microbial isogenic population (Lewis, 2010).

A subpopulation of persister cells is generated spontaneously in an actively growing population. This persister fraction can be modified by environmental stresses. This spontaneous phenotypic dichotomy is thought to result from redundant stress responses but includes stochastic variation in gene expression mediated by two gene toxin/antitoxin (TA) systems. When the toxin is in excess, the persister fraction in the bacterial population is increased (Maisonneuve et al., 2011). *B. pseudomallei* has several TA gene pairs and one has been demonstrated to play a role in determining the persister fraction in a bacterial population (Butt et al., 2013, 2014). A number of environmental stresses can modulate the level of persistence in an isogenic microbial population. These stresses include heat (Cardoso et al., 2010), oxidative stress and associated DNA damage (Dörr et al., 2009; Vega et al., 2012; Wu et al., 2012), cell membrane stress (Poole, 2012), quorum sensing signaling (Vega et al., 2012), carbon source transitions and nutrient stress (Nguyen et al., 2011), and cells in biofilms (Keren et al., 2004a).

*Burkholderia pseudomallei* causes a severe systemic infection named melioidosis. Melioidosis is endemic in tropical regions of South East Asia and northern Australia. Melioidosis can reemerge months to years after apparently successful antibiotic treatment (Cheng and Currie, 2005). Surviving melioidosis confers no immunity to subsequent challenge with the same agent nor are there vaccines that are protective against melioidosis (Brett and Woods, 2000). In particular, *B. pseudomallei* is a category B NIAID priority pathogen and is listed as a tier 1 HHS and USDA select agent (tier 1—presents the greatest risk of deliberate misuse with the most significant potential for mass casualties or devastating effects to the economy critical infrastructure, Choi et al., 2008).

Infections caused by *Burkholderia cepacia* complex bacteria are difficult to eradicate with antibiotics, especially in cystic fibrosis patients due to the rapid development of antibiotic resistance and/or evasion of treatment via the establishment of biofilms.

A major concern in the management of disease caused by these organisms is that an aggressive antibiotic regimen is required due to the inherent resistance of *Burkholderias* to aminoglycoside and macrolide antibiotics (Moore et al., 1999). In addition, an important feature of these diseases—especially melioidosis—is that recurrence is common even after completion of antibiotic treatment and apparent clearing of the bacteria, occurring in 3–25% of patients (Cheng and Currie, 2005; Hayden et al., 2012). Molecular typing indicates that most cases of relapse are due to recrudescence of the original infecting strain. Prolonged therapy has been shown to reduce relapse but the multi-drug aggressive therapy is associated with a high rate of adverse side-effects. A similar recurrent phenotype is observed in *Burkholderia cepacia* complex and *P. aeruginosa* infections in cystic fibrosis (Drevinek and Mahenthiralingam, 2011). Of importance to military health and biodefense are the risk of antibiotic failure and subsequent disease recurrence in melioidosis. While its etiologic agent, *B. pseudomallei*, poses a bioterrorism threat, it also poses a significant health risk for active duty personnel and veterans returning from endemic regions, especially the risk of late-onset melioidosis that can occur as late as 62 years after deployment (Cheng and Currie, 2005; Ngauy et al., 2005).

These recurrent and latent infections are likely caused by failure to eradicate the organism during an initial infection, which could in turn result from a non-replicating or slow-growing persister cell subpopulation that are tolerant to antibiotics. Some studies in other bacteria suggest that persister cells evade antibiotic therapy and/or the immune system either by forming a physical barrier (biofilm) or by developing reversible mutations that switch off the expression of non-essential genes (Percival et al., 2011; Keren et al., 2012; Lewis, 2012). Evidence largely supports that persister cells are metabolically dormant members of a vegetative population of metabolically active cells (reviewed in Wood et al., 2013). Persisters are thought to exist as dormant variants of bacterial cells in a growing population, a state generated by a stochastic process (Lewis, 2010; Mulcahy et al., 2010). Due to their slow or non-existent metabolism, bacterial persisters are tolerant of antibiotic exposure (Gefen and Balaban, 2009; Lewis, 2010), yet are not antibiotic resistant due to mutations. The SOS response (Dörr et al., 2009), genetic mutations (Moyed and Bertrand, 1983; Dörr et al., 2010), and even growth states (Keren et al., 2004a) are known to increase the level of persister cells in a population.

Several studies argue that the persister state is an active stress response of bacterial cells (Nguyen et al., 2011; Khakimova et al., 2013; Orman and Brynildsen, 2013). Perhaps in reality an active stress response is in play that ultimately achieves a persister state typically characterized by low levels of metabolic activity.

In this work we examined the persistence and emergence of tolerance to antimicrobials in several species of *Burkholderia* and in *P. aeruginosa* strain PA14. As a way of initiating a study of the regulatory infrastructure of the persister state in *B. pseudomallei*, we have undertaken experiments to identify environmental stressors that alter the persister fraction in a population of *Burkholderia pseudomallei* cells. We found that the persister fraction in *B. pseudomallei* cultures is modulated by environmental factors or an antibiotic challenge, and that they exhibit an extraordinarily high rate of drug tolerant persister cells in response to challenge by severe nutritional deprivation.

## Materials and methods

### Bacterial strains and culture conditions

*B. pseudomallei* Bp82 (Propst et al., 2010), a Δ*purM* mutant that is avirulent in immunocompetent and immunodeficient animals and is exempt from select-agent rules, was obtained from H. Schweizer. *B. cenocepacia* BC7, K56-2S, K56-2V (Varga et al., 2013) were obtained from J. Goldberg. *B. multivorans* CGD1 and CGD2 (Varga et al., 2012) were obtained from S. Holland. *B. thailandensis* E264 (Yu et al., 2006) was obtained from D. DeShazer. For *Burkholderia*, routine medium was Miller LB (Atlas, 2010) broth and agar. *Pseudomonas aeruginosa* PA14 (He et al., 2004) liquid medium was Miller LB (Atlas, 2010). Ciprofloxacin was obtained from MP Biomedicals (Aurora, Ohio, United States). Levofloxacin, Trimethoprim, Doxycycline, Gentamicin, and Cefotaxime were obtained from Sigma (St. Louis, MO). All bacteria were grown at 37°C in ambient air.

### Minimum inhibitory concentration determination

Minimum inhibitory concentrations (MIC) for antibiotics were determined by broth microdilution following standard NCCLS protocols (Institute, 2006) modified to use LB broth medium. In each case, the MIC was determined to be the concentration at which no visible growth was observed. The MICs for *B. pseudomallei* Bp82 in LB broth culture are: cefotaxime 4 μg/mL, ciprofloxacin 2 μg/mL, gentamicin 32 μg/mL, chloramphenicol 4.2 μg/mL, trimethoprim 4.7 μg/mL, and doxycycline 0.5 μg/mL.

### Persister fraction determination

The assay for determination of the persister fraction in a bacterial population was adapted from that of R.W. Titball (Hemsley et al., 2014) as follows. The bacterial culture being analyzed is pelleted by centrifugation and resuspended to a standard OD_600_ of 1 (~10^9^ CFU/mL) in fresh LB medium. The viable colony forming unit (CFU) concentration of the resuspended bacterial culture is obtained by serial dilution, plating, and counting of the colonies after 2 days at 37°C on LB agar plates. The fraction of drug tolerant cells within the resuspended population is determined by exposing the bacterial culture to antibiotics using a concentration of 100X the MIC for cefotaxime and 10X the MIC for ciprofloxacin and gentamicin employed in this assay. The bacterial culture in fresh medium at the above cell density was combined with the antibiotic and placed in wells (500 μl per well) of a 48 well flat bottom microtiter plate (Corning Costar 3548). The plate is incubated without shaking at 37°C for 24 h in an incubator with ambient air. At the end of the incubation period the samples were transferred to microcentrifuge tubes and centrifuged for 5 min at maximum speed in a bench top centrifuge to pellet the cells. The antibiotic containing supernatant was removed and the cells were resuspended in 500 μl fresh LB medium as a wash step. The cells are again pelleted and resuspended in 500 μl fresh LB medium. Viable CFU counts after the antibiotic challenge are obtained by serial dilutions, plating, and counting as for the total viable CFU count before the antibiotic challenge. The drug tolerant persister fraction was the number of cells that survived the antibiotic challenge divided by the number of cells placed into the well based on the pre-drug challenge viable CFU count. All assays were performed in biological triplicate. The persister fractions and CFU counts reported are the mean value of the three replicates. The error bars represent the standard deviation (SD) over the mean from at least three independent experiments. Antibiotic resistant cells were quantified by plating on solid medium under 50X MIC antibiotic selection.

### Growth in the presence of 0.25 X MIC levels of antibiotics

In order to explore the effect on the persister fraction in a population of *B. pseudomallei* Bp82 cells grown in liquid LB culture with shaking in the presence of sub-inhibitory concentrations of antibiotics, an overnight culture of Bp82 was used to inoculate 50 mL of LB broth in 250 mL Erlenmeyer flasks. The flasks contained 0.25 X MIC of one of the following antibiotics: chloramphenicol, trimethoprim, doxycycline, cefotaxime, and ciprofloxacin, or no antibiotic as the reference controls. The initial inoculum was such that three doublings were required to achieve the targeted final OD_600_ which for chloramphenicol, trimethoprim, and doxycycline was 0.1 and for cefotaxime, and ciprofloxacin was 2. The flasks were then incubated at 37°C in a shaker incubator at 200 rpm. The OD was monitored during growth and when the target OD was attained, a persister assay using a 100X cefotaxime challenge was performed as described. Each experiment was performed in biological triplicate.

## Results

### Determination and characterization of persister fraction within bacterial populations in liquid culture for *Burkholderia* spp. and *Pseudomonas aeruginosa*

Based on previous studies in *Burkholderia* spp. and *Pseudomonas aeruginosa* of disease recurrence (Drevinek and Mahenthiralingam, 2011) and extreme long term viability under nutrient restriction including distilled water for *B. pseudomallei* (Moore et al., 2008; Hamad et al., 2011), we hypothesized that members of the *Burkholderia* genus compared to *Pseudomonas aeruginosa* PA14 were likely to enter a persister state within a population of *in vitro* growing cells at mid-log phase growth at a high measurable frequency. We tested six different isolates of *Burkholderia cepacia* complex, *B. pseudomallei* and *B. thailandensis*, and *P. aeruginosa* to determine their ability and frequency of entry into a persister state as assayed by tolerance to a high levels antibiotic challenge. Exponentially growing cells were exposed to levofloxacin (a fluoroquinolone) at 10-fold (10X) the MIC and persister cells were enumerated as those that were viable after at least 16 h post-antibiotic treatment (Mulcahy et al., 2010). All tested *Burkholderia* spp. had between 0.05 and 0.25% of the population as persister cells—a rate that was at least 100–500 times higher than wild-type *P. aeruginosa* PA14 (Table 1). The rates for *Burkholderia* species were comparable to the *P. aeruginosa* high persister phenotype as described (Mulcahy et al., 2010). In order to ascertain whether these cells were viable because of antibiotic resistance, and not due to tolerance, antibiotic-exposed cultures were plated in both selective (50X MIC) and non-selective media for enumeration. Fewer than 1 in 10^8^ cells of the antibiotic-tolerant cells grew under selection, demonstrating a very low rate of conventional antibiotic resistance.

**Table 1:** **Rates of persisters for *Burkholderia* spp. and *Pseudomonas aeruginosa* in liquid culture mid-log growth assayed by challenge with 10X MIC levofloxacin**.

**Species and strain**	**MIC (μg/mL)**	**Persister rate (%)**
*B. multivorans* CF1	16	0.085
*B. multivorans* CF2	16	0.5
*B. multivorans* CGD1	16	0.26
*B. multivorans* CGD2	16	0.25
*B. cenocepacia* K56-2V	4	0.19
*B. cenocepacia* K56-2S	4	0.05
*B. cenocepacia* BC7	16	0.02
*B. thailandensis* E264	4	0.14
*B. pseudomallei* Bp82	4	0.11
*P. aeruginosa* PA14	0.25	0.0007

### The time dependent killing of *B. pseudomallei* BP82 under high antibiotic dosage challenge reveals a time interval with sufficient viable CFU stability for enumerating the persister fraction of a population

**Figure 1:**
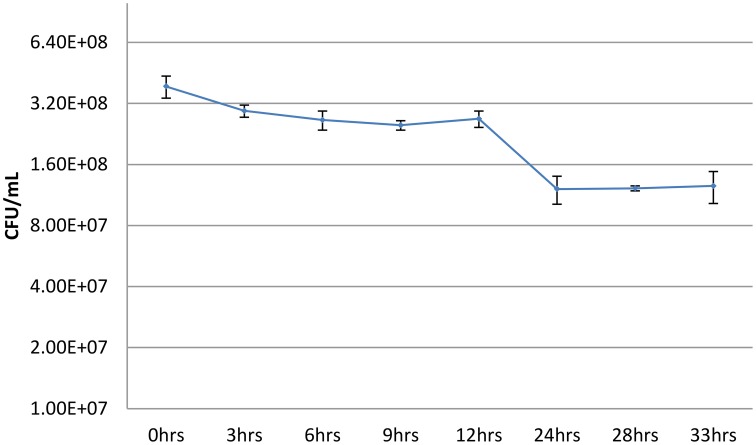
**Viable Bp82 CFUs vs. duration of exposure to 100X MIC cefotaxime without shaking**. Actively growing shaking cultures of Bp82 in liquid LB medium at 37°C were allowed to attain an OD_600_ of 1.4. The cells were pelleted by centrifugation and resuspended to an OD of 1 in fresh LB medium. The total viable CFU/mL concentration of the population was determined by serial dilution and plating (0 h). Aliquots, 0.5 mL, of the culture were placed into multiple wells of a 48-well flat bottom microtiter plate with cefotaxime at 100X MIC (400 μg/mL) and the plate was placed in a 37°C incubator for the indicated times without shaking. The contents of triplicate wells were evaluated for the remaining surviving bacteria as determined by serial dilution and plating at each indicated time point. The figure represents the results of three biological replicates. The error bars represent the standard deviation (SD) over the mean from at least three independent experiments.

The goal of our study is to identify some environmental factors that influence the persister fraction within a *B. pseudomallei* Bp82 population. The assay for determination the persister fraction in a bacterial population was adapted from that of R.W. Titball (Hemsley et al., 2014). The fraction of the cells in the persister state within a population is determined by the dilution and plating of the cells in the population to obtain a total vial CFU count per mL of culture followed by determining the viable CFU count per mL that survives a high dose antibiotic challenge. The persister fraction is calculated by calculating the fraction of the total viable population that survives the antibiotic challenge. The assay uses a 100X MIC dose of cefotaxime on bacterial cells resuspended in fresh LB medium at an OD_600_ = 1, thus assuring that the cells are not in stationary phase. While the details of the assay are described in Materials and Methods, we needed to establish the duration of the antibiotic challenge for Bp82 so we determined a time course of the viable CFU decline under the antibiotic challenge under the conditions of the assay. As shown in Figure 1, after the initial decline in the CFU viability due to the antibiotic killing of the non-persister members of the bacterial population, the CFU viability is stable from 24 h of antibiotic exposure until at least 33 h.

**Figure 2:**
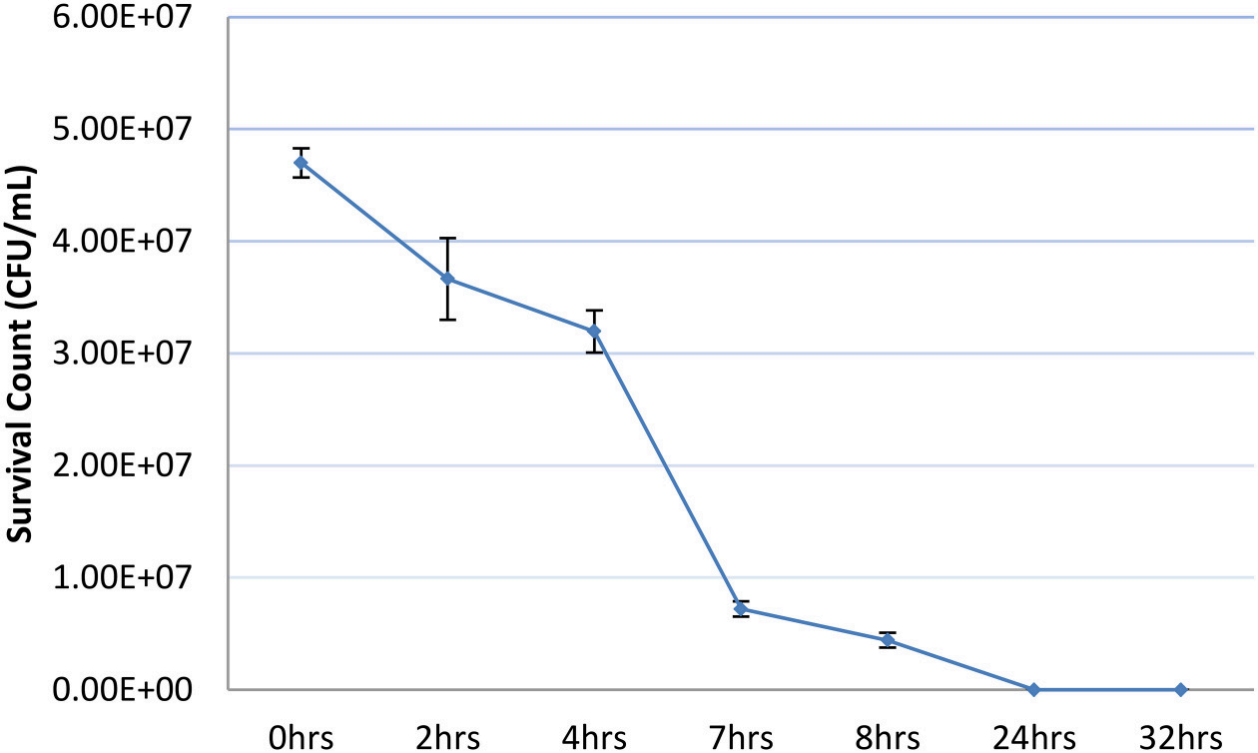
**Viable Bp82 CFUs vs. duration of exposure to 100X MIC cefotaxime with shaking**. Actively growing shaking cultures of Bp82 in liquid LB medium at 37°C were grown to attain an OD_600_ of 0.1. The total viable CFU/mL concentration of the population was determined by serial dilution and plating (0 h). Cefotaxime was added to 100X MIC (400 μg/mL) and the culture was returned to shaking at 37°C. At the indicated times, 0.5 mL aliquots were removed to determine the viable CFU/mL concentration by dilution and plating after removal and replacement of the antibiotic containing medium. The figure represents the results of three biological replicates. The error bars represent the standard deviation (SD) over the mean from at least three independent experiments.

The persister cefotaxime challenge assay we use based on the findings shown in Figure 1 accomplishes the antibiotic challenge in microplate wells without shaking. We modified the antibiotic challenge format by growing shaking cultures of Bp82 in LB liquid medium to an OD_600_ of 0.1 and removing an aliquot for total viable CFU determination followed by the addition of cefotaxime to a concentration of 400 μg/mL. Shaking was continued at 37°C and aliquots were taken at the times indicated in Figure 2 to obtain the surviving CFU/mL count at that time point during the antibiotic challenge. As shown in the figure, in contrast to a surviving persister population surviving antibiotic challenge after 24 h without shaking (Figure 1), when the antibiotic challenge is accomplished in a shaking culture, no viable bacterial cells remain at this time point. The most obvious difference in the two challenge conditions is the limitation of oxygen available to the cells not being shaken. Hemsley et al. (2014) reported that drug-tolerant Burkholderia cells are characterized by an anaerobic metabolic signature. It is likely that the ready availability of oxygen in the shaking culture resulted in pulling the initial persister population from this state over the 24 h challenge time course resulting in antibiotic killing of the entire population. The shake culture experiment was repeated using a 10X gentamicin challenge. A similar profile of the CFU decline was observed with no viable cells remaining at 24 h into the challenge (data not shown).

### The use of different antibiotics in the persister challenge assay enumerates a different fraction of the population as persisters

**Figure 3:**
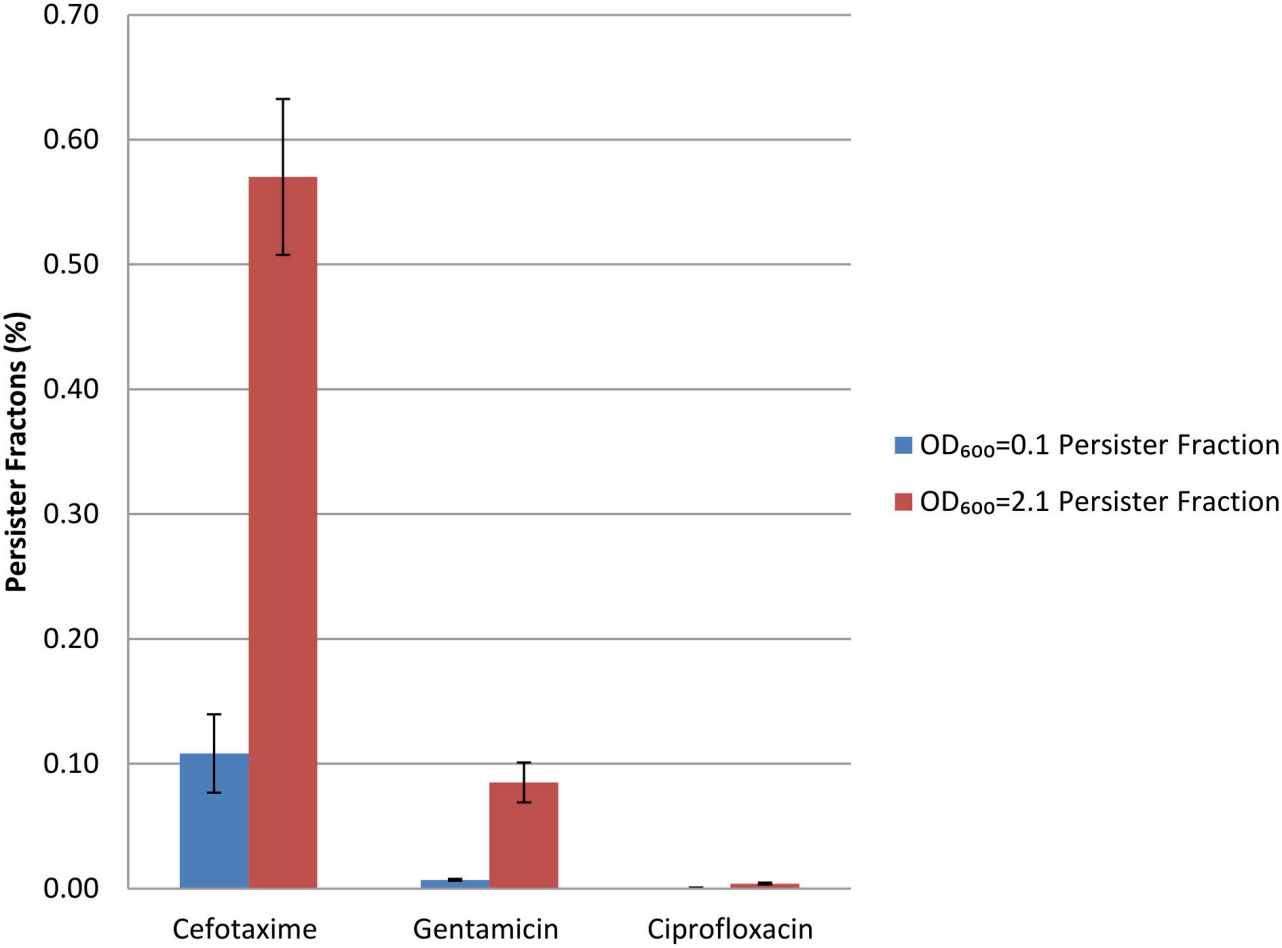
**Persister fractions determined at two OD_600_ growth states using three different antibiotics for persister selection**.

We then tested whether antibiotic tolerance in *B. pseudomallei* Bp82 was independent of the antibiotic used in the persister assay and could thus potentially reemerge after treatment with clinically-relevant antibiotics. To this end, we performed persister assays using 100X cefotaxime and 10X ciprofloxacin and gentamicin. The assays were conducted at two different points in the growth curve, one at an OD_600_ of 0.1 and the other at an OD_600_ of 2.1. The observed persister fraction at each of the two time points was replicated using all three of the antibiotics in the persister assay (Figure 3).

Challenge with 100X MIC cefotaxime revealed a persister fraction of 0.11 and 0.57 at OD_600_ of 0.1 and 2.1 respectively; with 10X MIC gentamicin the corresponding persister fractions were 0.007 and 0.085; and with 10X MIC ciprofloxacin they were 0.00059 and 0.004. The lower frequency of drug tolerant persisters revealed by challenge with ciprofloxacin has been previously reported (Hemsley et al., 2014). Two observations are of note relative to these data. The first is that the persister fraction is higher at the later growth phase point, OD_600_ = 2.1, than at the earlier point of OD_600_ = 0.1. The second is that depending on the antibiotic used in the challenge, the fraction of persisters in the populations analyzed is quite different, suggesting that each antibiotic is selecting a different but likely overlapping persister subpopulation.

### The persister fraction is influenced by some antibiotics

While not directly known to alter the rate of persisters within a bacterial population, bacterial antibiotic therapy alone has been shown to increase the rate of spontaneous mutations (Kohanski et al., 2010). Bacteria exposed to sub-inhibitory doses of antibiotics have been demonstrated to exhibit mutations through production of reactive oxygen species and through activations of the error-prone DNA polymerase PolIV (Gutierrez et al., 2013; Nair et al., 2013). Resistance mutations can be selected under such minimal drug pressure (Gullberg et al., 2011). We have undertaken an evaluation of the potential for antibiotics employed in melioidosis therapy (Schweizer, 2012) to alter the persister rate in *in vitro* culture populations of Bp82. The antibiotics we employed in the study are chloramphenicol, trimethoprim, doxycycline, ceftazidime and ciprofloxacin. We have determined the Bp82 MIC for these antibiotics by broth microdilution following standard NCCLS protocols (Institute, 2006). These MICs are provided in Materials and Methods.

We targeted an early log phase growth point (OD_600_ = 0.1) for 0.25X MIC chloramphenicol, trimethoprim, and doxycycline cultivation and at late log phase point (OD_600_ = 2) for cefotaxime and ciprofloxacin cultivation in LB broth shaking cultures at 37°C for evaluating the consequence on the persister fraction of cultivation in the presence of the sub-inhibitory levels for each of the antibiotics tested. Briefly, an overnight culture of Bp82 grown in the presence and absence of the antibiotic under consideration was diluted in the morning to require three doublings in the presence of antibiotics before initiating the standard cefotaxime persister challenge assay described in Materials and Methods. No-antibiotic controls were treated in the same way. Growth was monitored by OD reading until the target ODs were attained. The persister fractions obtained with and without antibiotic were compared to quantify the consequence, if any, for the cultivation in the presence of the antibiotic. As before, all experiments were performed in triplicate. While cultivation in the presence of the 0.25 X MIC levels of chloramphenicol did not influence the resulting persister fraction, the trimethoprim cultivation greatly increased the persister fraction while cultivation in the presence of doxycycline, cefotaxime, and ciprofloxacin resulted in persister fractions lower that that observed in the absence of the antibiotic (Table 2).

**Table 2:** **Cultivation of *B. pseudomallei* in the presence or absence of 0.25 X MIC of listed antibiotics**.

**Antibiotic**	**Bp82 + Antibiotic Persister fraction**	**Bp82 − Antibiotic Persister fraction**
Chloramphenicol	0.11	0.1
Trimethoprim	0.32	0.07
Doxycycline	0.03	0.08
Cefotaxime	0.31	0.43
Ciprofloxacin	0.18	0.43

### Environmental factors that influence the *In vitro* persister population

To understand whether growth conditions affected *B. pseudomallei* Bp82 persister cell induction, we studied the fraction of cefotaxime tolerant cells (at 100X MIC) as a function of growth stage. As noted earlier, we found that the fraction of the population exhibiting the persistence phenotype fraction increased as the growth cycle progressed through the log phase and into the stationary phase of growth (Figure 3). This growth phase dependence of the persister fraction in Bp82 is very similar to that reported for *Burkholderia thailandensis* (Hemsley et al., 2014). The antibiotic tolerance phenotype in *B. pseudomallei* was not due to increased antibiotic resistance, as cells plated on selective media containing 20X MIC cefotaxime after surviving the persister assay antibiotic challenge did not grow. Our results showed that antibiotic tolerance and persistence in *B. pseudomallei* Bp82 exhibits a similar phenotype to the persister phenotype observed in a fully virulent *B. pseudomallei* strains (Butt et al., 2013).

### Environmental factors that maximize the persister fraction

#### Late stationary growth phase

*B. pseudomallei* Bp82 was cultured in LB broth to the late stationary growth phase to determine the maximum persister fraction that could be obtained during LB liquid culture. Cultures were established in triplicate using 50 mL conical centrifuge tubes with a 10 mL broth culture volume with shaking at 37°C. The cultures were assayed for persister fraction starting from day 5 to as late as day 15. Daily assays were performed from days 10 to 15. The maximum persister fraction attained was 0.65 at day 13 when the viable CFU count had declined to 5.2 × 10^6^ per mL (Table 3).

**Table 3:** **Evaluation of persister fraction during very extended LB broth culture**.

**Cultivation day**	**CFU/mL before selection**	**CFU/mL after selection**	**Persister rate**
5	6.8 E 8	3.3 E 7	0.05
10	1.3 E 9	2.3 E 7	0.018
11	2.2 E 8	4.6 E 6	0.021
12	1.6 E 8	7.6 E 7	0.48
13	5.2 E 6	3.4 E 6	0.65
14	2.8 E 5	1.3 E 5	0.46

#### Distilled water storage

**Figure 4:**
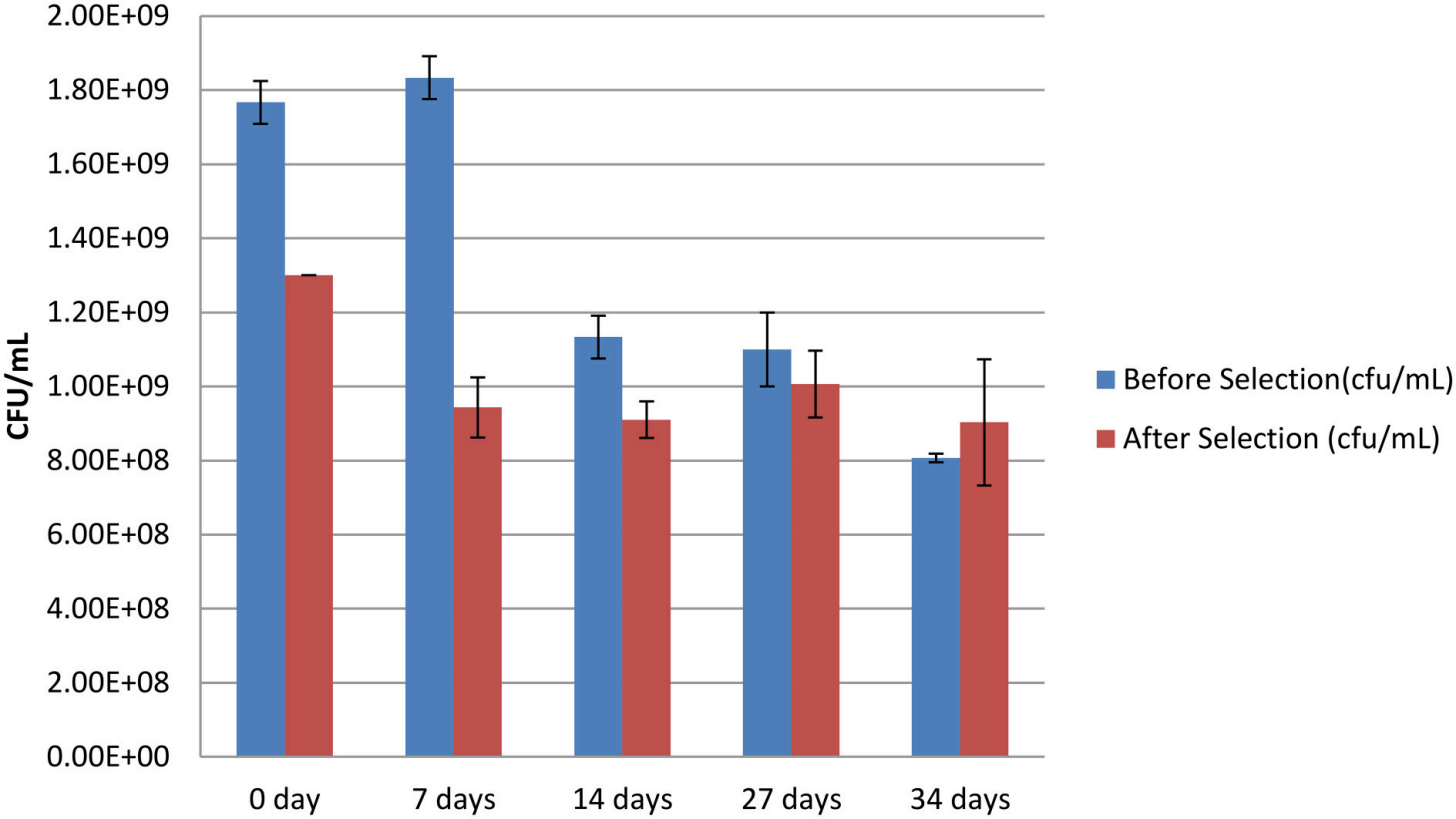
**Viable colony forming units (CFU) after storage in distilled water Blue is viable cells before antibiotic persister selection and red is viable cells after antibiotic persister selection**. The error bars represent the standard deviation (SD) over the mean from at least three independent experiments.

*B. pseudomallei* is reported to survive in distilled water storage for 16 years (Pumpuang et al., 2011). Expression profiling and mutant analysis of *B. pseudomallei* in distilled water storage was used to explore genes essential for long term water survival (Moore et al., 2008). In investigating environmental factors that may maximize the fraction of persisters in the population we determined the persister fraction present over time in a population of Bp82 maintained in distilled water at ambient room temperature with shaking. An overnight culture of *B. pseudomallei* Bp82 colony was used to inoculate triplicate 10 mL of LB broth in 50 mL conical centrifuge tubes which were then grown overnight with shaking, achieving an OD_600_ of 1.8. The three bacterial cultures were pelleted by centrifugation and the pellets were resuspended in 10 mL distilled water for washing. The bacteria were again pelleted by centrifugation and again suspended in 10 mL distilled water in each of the three tubes. The cultures were placed at room temperature with shaking. Persister assays were performed at intervals to determine the change in persister fraction in the cultures over time under this storage condition. The findings are shown in Figure 4. While the level of viable cells declined over the period of observation, the fraction of persisters increased achieving a level of 100% persisters in the population at 34 days.

## Discussion

Pathogenic bacterial populations contain a fraction of the isogenic cells in a state of dormancy that allows them to be tolerant to a challenge by multiple antibiotics while not being resistant to the antibiotics through genomic changes conferring resistance. The drug tolerance associated with this persister state is achieved through the lack of metabolic activity of the bacterial target of the antibiotic. For example, in the absence of cell wall synthesis in a dormant persister cell a β-lactam antibiotic will not kill the cell since the killing is dependent upon inhibition of the cell wall synthesis during cellular replication. In stationary phase cells of *P. aeruginosa, Escherichia coli*, and *Staphylococcus aureus* the fraction of the population in the persister state can achieve a maximum level of ~0.01 (Keren et al., 2004b). In the present study, during mid-log growth several *Burkholderia cenocepacia* complex species achieved a persister fraction between 25 and 50% revealed by levofloxacin challenge, while the *B. pseudomallei* Bp82 fraction was 11% and the *P. aeruginosa* PA14 fraction was 0.07% (Table 1). As observed for the ciprofloxacin persister challenge, fluoroquinolone antibiotics give a lower persister fraction in the assay than the several other antibiotics (Figure 3). These observations indicate that some pathogenic members of the Burkholderia genus can achieve very high persister fraction in mid-log phase culture relative to *E. coli, S. aureus*, and *P. aeruginosa*. The association between the *in vitro* high persister fraction potential of *B. pseudomallei* and the high rate of reemergent infections in melioidosis patients after an antibiotic regimen resulting in an apparent cure provides justification for pursuing *in vivo* studies for demonstrating a causal relationship between bacterial drug tolerant persistence, and latent and reemergent melioidosis episodes.

We have used five antibiotics in conjunction with persister assays to determine if exposure to sub-inhibitory level of antibiotics influences the persister fraction in a population of Bp82 cells. These antibiotics are cefotaxime, a β-lactam inhibitor of cell wall syntheses; chloramphenicol, a protein elongation inhibitor of protein synthesis; doxycycline, a tetracycline class protein synthesis inhibitor that that blocks the attachment of the charged aminoacyl-tRNA to the ribosome mRNA complex; trimethoprim, a dihydrofolate reductase inhibitor that blocks the thymidine synthesis pathway thus inhibiting bacterial DNA synthesis; and ciprofloxacin, a fluoroquinolone that inhibits DNA gyrase thus blocking bacterial DNA replication and cell division. Cultivation of Bp82 in the presence of 0.25X MIC ciprofloxacin results in a ~2X lower persister fraction than in the absence of the antibiotic (Table 2), supporting the hypothesis that ciprofloxacin exposure may reduce the ability of *B. pseudomallei* to remain in the persister state or speeds up the transition from the dormant persister to the vegetative growth state. At 0.25 MIC exposure to growing Bp82 cells, the persister fraction is also reduced for cefotaxime and doxycycline while the persister fraction is much higher in the presence of trimethoprim (Table 2). Cultivation in chloramphenicol does not alter the persister fraction from that observed in its absence. Perhaps these persister fraction altering antibiotics cause changes in the kinetics of the persister-vegetative cell transitions, thus altering the equilibrium persister fraction in the population.

The additional topic of focus for the current study is to explore conditions that may lead to the attainment of a maximum level of persisters in an *in vitro* population of Bp82. We first directed our effort to extended maintenance of stationary phase cultures of Bp82 in LB medium at 37°C with shaking. As shown in Table 3, the population of viable cells is in decline for the analyzed 5th through 14th day of culture. A maximum persister rate of 65% is attained on day 13.

We next turned our attention to determining the level of persisters in the population of Bp82 stored in distilled water. As noted earlier, *B. pseudomallei* is notable for its ability to survive long term storage in distilled water. Our time course in distilled water was conducted with shaking at room temperature. As shown in Figure 4, although the viable CFU level in the distilled water declines continuously during the 7–49 day period, the absolute level of persisters is stable until the day 34 assay. Considerable decay in the absolute persister population occurs between days 34 and 49 (data not shown), although all of the cells remaining viable in this time window are persisters. Clearly being in the persister state provides survival advantage to Bp82 cells under the nutritional and osmotic stress of storage in distilled water. Perhaps this high level of persister formation is a response in *B. pseudomallei* to severe stress analogous to the environmental stress induced initiation of sporulation in some other bacterial species.

We have identified conditions for obtaining high levels of persisters in *B. pseudomallei* during *in vitro* culture/storage. We have also characterized the consequence on the persister fraction in a *B. pseudomallei* population by *in vitro* cultivation with sub-inhibitory levels of 5 antibiotics with different bacterial mechanisms of action. Additional studies can now be undertaken with *B. pseudomallei* populations composed almost entirely of drug tolerant persister cells for elucidating the genomic infrastructure that regulates the transitions between the vegetative growing population members and the dormant persister members of a culture population. Information on this issue will provide valuable background before proceeding to determine if a causative relationship exists between latent and recurrent *B. pseudomallei* infections and the potential *in vivo* persister status of *B. pseudomallei* facing a host immune system challenge coupled with a therapeutic antibiotic intervention. Once causality is established, the persister infrastructure can be targeted for drug intervention to suppress the formation of persisters or to eliminate persisters from the *B. pseudomallei* population causing melioidosis in a mammalian or human host, thus reducing or eliminating recurrent infections.

### Conflict of interest statement

The authors declare that the research was conducted in the absence of any commercial or financial relationships that could be construed as a potential conflict of interest.
